# Rasgos antropométricos craneofaciales de interés odontológico forense en la estimación de sexo, grupo étnico y edad. Revisión de la literatura

**DOI:** 10.21142/2523-2754-0901-2021-047

**Published:** 2021-03-11

**Authors:** José Alberto Castillo-Páez, Luis Guillermo Villasmil-Suárez, Natacha Valentina Guada-Melet

**Affiliations:** 1 Departamento de Estomatoquirúrgica, Facultad de Odontología de la Universidad de Carabobo. Valencia, Venezuela. josecastillo031285@gmail.com, luisguillermovillasmil@gmail.com Universidad de Carabobo Departamento de Estomatoquirúrgica Facultad de Odontología Universidad de Carabobo Valencia Venezuela josecastillo031285@gmail.com luisguillermovillasmil@gmail.com; 2 Departamento de Prostodoncia y Oclusión, Facultad de Odontología de la Universidad de Carabobo. Valencia, Venezuela. natachaguada@hotmail.com Universidad de Carabobo Departamento de Prostodoncia y Oclusión Facultad de Odontología Universidad de Carabobo Valencia Venezuela natachaguada@hotmail.com

**Keywords:** rasgos craneofaciales, antropología forense, odontología forense, Skull-Facial Features, Forensic Anthropology, Forensic Dentistry

## Abstract

Una de las funciones principales del antropólogo y del odontólogo forense es la de, mediante métodos no rutinarios, dar con la identidad de un sujeto gracias a sus características físicas observables. Esas características, especialmente las óseas, permiten al profesional identificar el sexo, la edad y la incidencia racial de los restos u osamentas que puedan hallar. El odontólogo centra su atención en rasgos pertenecientes al sistema estomatognático, mientras que el antropólogo estudio el conjunto de huesos que componen de forma general el cuerpo humano. En este estudio, se busca exponer los rasgos antropométricos craneofaciales propios del estudio del antropólogo para estimar sexo, grupo étnico y edad, que son de interés odontológico y útiles para la identificación de restos óseos u osamentas. Se analizan las investigaciones realizadas para la determinación del sexo, la incidencia racial y la edad, además de un aporte acerca de la denominada antropología dental, que estudia los elementos propios del sistema estomatognático desde el punto de vista antropológico. Se revisó literatura electrónica mediante buscadores como PubMed, Google Académico y SciELO, con las palabras “ForensicDentistry”, “ForensicAnthropology”, “Sex Determination”, “Ancestry Determination” y “Age Determination”. A partir de lo hallado, se concluyó que es de gran importancia para el odontólogo el conocimiento de los rasgos antropológicos craneofaciales desde el punto de vista forense para la individualización, así como la significancia de la interdisciplinariedad entre el trabajo del odontólogo y el del antropólogo para la labor de identificación.

## Abstract (en)

One of the main functions of anthropologists and forensic dentists is to identify subjects according to physical characteristics of the features presented using non-routine methods. These characteristics, especially bone, allow professional to determine the sex, age and racial origin of the remains or skeleton found. Dentist focus on features belonging to the stomatognathic system, while anthropologists study the bones that make up the human body. In this study, we describe the investigations of cranio-facial anthropometric features of dental interest carried out by anthropologists to identify the sex, race and age of skeletal remains or skeletons. in addition, we describe the so-called dental anthropology that studies the elements of the stomatognathic system from an anthropological point of view. Electronic literature was reviewed through search engines such as PUBMED, Google Academic and SciELO with the search terms “Forensic Dentistry, Forensic Anthropology, Sex Determination, Ancestry Determination and Age Determination”. Knowledge of the craniofacial anthropological features from the forensic point of view is of great importance in individualized and multidisciplinary studies by forensic dentists and anthropologists in the identification of skeletal remains.

## INTRODUCCIÓN

La identificación se define como el proceso mediante el cual se establecen los atributos de un individuo. Cuando existen solo restos humanos, como osamentas o restos óseos, este proceso puede hacerse bastante difícil ^1^. En esos casos, entra en acción el odontólogo forense, cuya labor consiste en comparar perfiles dentales *ante* y *post mortem* para determinar los atributos individuales y así lograr la identificación. Esto es posible gracias a que las unidades dentarias resisten totalmente la descomposición y soportan relativamente bien las condiciones ambientales extremas. Además, se ha demostrado que cada persona posee características dentales individuales que la hacen esencialmente única y reconocible ^2^.

La identificación odontológica y antropológica forense se realiza en caso de muertes extremas y repentinas, como las causadas por explosiones, incendios y accidentes de tránsito, o cuando se hallan restos humanos descompuestos, que requieren un análisis metodológico específico, desde el punto de vista médico legal, para conocer la identidad del individuo ^3^.

Por una parte, la identificación antropológica considera la morfología de los huesos desde un punto de vista macro. De hecho, los estudios antropométricos son aquellos que hacen referencia a la cuantificación de la forma, es decir, las medidas de segmentos corporales, los índices proporciones y los ángulos de estructuras anatómicas, con el fin de individualizarlos y que estos, a su vez, brinden información sobre el sexo, la edad, la afinidad racial, la estatura, las asimetrías u otras particularidades que sean útiles para el proceso de identificación. Por ejemplo, para estimar la edad cronológica al momento de la muerte, básicamente, se considera el crecimiento o los cambios observables en la clavícula, la pelvis y la cuarta costilla ^4^^,^^5^.

Por otro lado, la odontología forense se ocupa del manejo de la evidencia dental, desde su correcta evaluación hasta su presentación al sistema de justicia. Esta evidencia no solo está constituida por las unidades dentarias, sino por todos los elementos del sistema estomatognático, que incluye la boca, la lengua, el maxilar, la mandíbula, la faringe y otras estructuras relacionadas con la masticación, la deglución y el habla ^6^^,^^7^.

Existen muchos elementos propios del estudio del antropólogo que se encuentran dentro del campo de acción del odontólogo forense, esencialmente los rasgos craneofaciales; por ello, mediante esta revisión, se busca exponer los rasgos antropométricos craneofaciales propios del estudio del antropólogo para estimar sexo, grupo étnico y edad, y que son de interés odontológico y útiles para la identificación de restos óseos u osamentas.

La importancia del conocimiento de los rasgos anatómicos craneofaciales es vital para el desarrollo de la actividad odontológica, esencialmente la forense, pues estos orientan tanto al odontólogo como al antropólogo forense para la estimación del grupo étnico, el sexo y la edad. Por ello, es de gran relevancia para el odontólogo conocer de qué forma aquellos rasgos que componen la anatomía craneofacial sirven a las ciencias forenses para la labor de identificación.

## MATERIALES Y MÉTODOS

Esta investigación se llevó a cabo utilizando recursos electrónicos obtenidos mediante la búsqueda en la web en buscadores como PubMed, Google Académico y SciELO, con las palabras “Forensic Dentistry”, “Forensic Anthropology”, “Sex Determination”, “Ancestry Determination” y “Age Determination”. Para la búsqueda, se consideraron factores como textos completos, textos en PDF y fecha de publicación, que comprendió datas recientes previas al desarrollo de la presente investigación. Los criterios de inclusión considerados para los artículos fue que guardaran relación directa con el objetivo de la investigación, es decir, exponer las actualizaciones en el análisis de los rasgos antropométricos craneofaciales propios del estudio del antropólogo, que son de interés odontológico. Los criterios de exclusión fueron cartas al director, tesis, periódicos, conferencias, noticias, comentarios y editoriales. Con ello se llegó, finalmente, a los 37 artículos que componen las referencias en la presente revisión.

### El análisis antropométrico de rasgos craneofaciales

La antropometría, como disciplina, busca resolver el “dilema” de la identificación mediante el análisis cuantitativo de las osamentas o restos óseos, es decir, según la medida de cada hueso, determinar si efectivamente se trata de un hueso humano, definir de cuál se trata y a qué género o grupo etario pertenece. Específicamente, en el caso del estudio de los huesos que integran el sistema estomatognático, los maxilares y craneales pueden aportar información clave para identificar las características mencionadas anteriormente, además de que son los que definen la morfología dentaria y la oclusión del individuo ^8^^,^^9^.

Tradicionalmente, el análisis antropométrico de estructuras óseas craneofaciales para la estimación de la edad consiste en el estudio de la osificación, el desarrollo y el grado de obliteración de cierres y suturas craneales, mientras que, desde el punto de vista odontológico, se realiza mediante los métodos que analizan la erupción dental, como el de Nola. Adicionalmente, la edad puede establecerse mediante el cálculo de los índices cefálicos, como el índice de altura craneal, el índice de ancho del cráneo, el índice orbitario, el índice gnático y el índice nasal. Por otro lado, la afinidad racial y la estimación del sexo pueden establecerse analizando las características morfológicas del cráneo, como forma y tamaño, configuración de fosas nasales, espina nasal, órbitas y suturas craneales ^9^^,^^10^.

### Estimación del sexo

Tradicionalmente, la estimación del sexo por rasgos antropométricos craneofaciales se realiza mediante la observación de la morfología del cráneo y la mandíbula. Aproximadamente, al 96% de las osamentas o restos óseos hallados se les puede estimar el sexo a partir de los datos obtenidos mediante la visualización de las diferentes características de estas estructuras óseas ^(11, 12)^. Las principales diferencias que se presentan para el dimorfismo sexual en el cráneo y la mandíbula pueden apreciarse de forma sintetizada en la Tabla 1.

**Tabla 1:** Diferencias de morfología craneal entre el sexo masculino y el femenino (modificado de Nagare et al.)

Rasgo	Masculino	Femenino
*Tamaño del cráneo*	Grande	Pequeño
*Arquitectura craneal*	Escabrosa	Suave
*Masa craneal*	Profunda	Menos profunda
*Cresta temporal*	Más prominente	Menos prominente
*Margen suborbital*	Redondo y grueso	Afilado
*Hueso cigomático*	Más pronunciado	Menos pronunciado
*Mandíbula*	Cuadrada	Redonda
*Arco superciliar*	Largo y pronunciado	Más pequeño
*Gonion*	Acampanado	Menos acampanado
*Dientes*	Alargados	Más pequeños
*Proceso mastoideo*	De medio a grande	De pequeño a medio
*Cavidad nasal*	Alta, de márgenes delgados	Baja, de márgenes amplios y redondeados
*Ángulo goniaco mandibular*	Obtuso	Obtuso hacia Recto
*Glabela*	Protrusa	Plana
*Eminencia mentoniana*	Cuadrada y alta	Triangular y baja
*Prominencias parietales*	Desarrolladas	No tan desarrolladas
*Protuberancia occipital externa*	Desarrollada	No tan desarrollada

En la actualidad, la estimación del sexo se realiza mediante herramientas como las radiografías panorámica y cefálica lateral, y la tomografía computarizada de haz cónico. Un ejemplo de esto es el estudio realizado por Nuzzolese et al. ^13^, en el que se utilizaron técnicas de geometría morfogénica para cuantificar la variación en la forma del hueso mandibular, es decir, el cuerpo y la rama ascendente de la mandíbula, lógicamente determinando su potencial con fines forenses identificativos. Para esto, se evaluaron 100 radiografías panorámicas, 50 de pacientes masculinos y 50 de pacientes femeninos, y se seleccionaron ciertos puntos anatómicos para establecer marcadores geométricos, realizar mediciones y establecer las diferencias notables en la morfología. Los puntos elegidos fueron el infradentario, el punto más superior del cóndilo mandibular, el punto más anterior del cóndilo mandibular, la línea que se forma a lo largo del borde inferior del cuerpo mandibular desde gnation a gonion, y la línea que se forma a lo largo del borde posterior de la rama ascendente desde el gonion hasta el punto más posterosuperior del cóndilo mandibular.

En suma, en el estudio se halló que existen diferencias significativas entre la forma de la mandíbula de un hombre y la de la mandíbula de una mujer, lo que indica un potencial bastante significativo del análisis radiográfico del hueso mandibular para la determinación del sexo, además de considerarlo un método estandarizado para diferenciar el dimorfismo sexual en el proceso de identificación.

**Figura 1:**
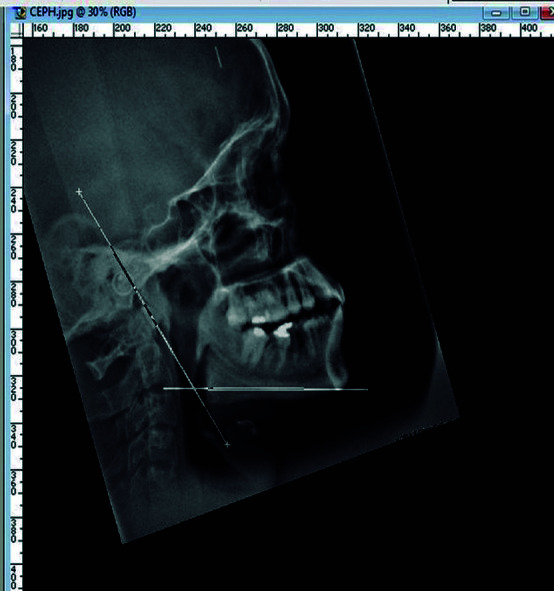
Línea horizontal en el borde inferior del cuerpo de la mandíbula y línea tangente a través del borde posterior de la rama (tomado de: Belaldavar et al.).

En otro estudio, Belaldavar et al. ^14^ quisieron determinar si el ángulo goniaco era de utilidad para la determinación de sexo en una población india. Se evaluaron 304 radiografías cefálicas laterales de 155 de pacientes femeninos y 149 de pacientes masculinos entre 18 y 30 años. Se midió el ángulo goniaco con el programa Adobe Photoshop CS3 (Adobe Systems Inc., Mountain View, EE. UU.) y se utilizaron procedimientos que arrojaron datos bastante certeros sobre el valor numérico del ángulo (Figura 1).

Así, del estudio, se infirió que existe una variabilidad estadística significante en la población india de hombres y mujeres, pues el ángulo goniaco femenino mide, en promedio, 122,7° y el masculino, 121,1°. Esta diferencia podría ayudar a determinar el sexo mediante una radiografía cefálica lateral.

En una visión un poco más actual, Albalawi et al. ^15^ consideran que la mandíbula es uno de los huesos más confiables al momento de determinar el sexo y, en concordancia, buscan establecer medidas mandibulares utilizando la tomografía computarizada de haz cónico. Estudiaron un total de 200 tomografías a las que les realizaron un análisis morfométrico de los ángulos formados por la intersección de las líneas que van de derecha a izquierda desde gonion hasta el mentón, es decir, se evaluó geométricamente la distancia lineal de gonion derecho al mentón, la distancia lineal del gonion izquierdo hacia el mentón y la distancia lineal de gonion derecho a gonion izquierdo.

De este análisis, llegaron a la conclusión de que la forma, las medidas y los ángulos de las tres líneas tienen diferencias resaltantes entre el sexo masculino y el femenino, por lo que puede ser un indicador potencial para determinar el sexo.

En suma, el análisis radiográfico, bien sea mediante una radiografía panorámica, una radiografía cefálica lateral o una tomografía computarizada de haz cónico, provee detalles morfológicos y estructurales del cráneo que pueden ser de utilidad para la estimación del sexo, al ser analizados con técnicas morfométricas aplicadas por el antropólogo forense ^16^.

### Estimación del grupo étnico 

Para hablar de estimación del grupo étnico, origen geográfico, afiliación o fenotipo racial mediante estructuras craneales, tradicionalmente, se empieza por los tres principales grupos poblacionales: caucasoide, negroide y mongoloide. Cada uno de ellos posee características estructurales y morfológicas craneales únicas que los distinguen. Pero, actualmente, la determinación de la raza podría tornarse complicada debido a la variedad incalculable de fenotipos raciales que se han generado gracias a la mezcla entre estos tres grupos étnicos ^17^.

Por ello, para la estimación del grupo étnico, se evalúan hoy rasgos propios de cada nacionalidad y origen étnico, considerando así los principales índices craneales:

- a. Cráneos dolicocefálicos. De forma ovalada y alargados, característicos del grupo poblacional negroide.
- b. Cráneos mesocefálicos. Medianos y proporcionados, propios del grupo poblacional caucasoide.
- c. Cráneos braquicefálicos. Redondeados y cortos, propios de grupos poblacionales mongoloides ^18^.

Dentro de este marco, estudios como el de Woo et al. ^19^, que quisieron examinar el índice craneal en la población moderna tailandesa, demostraron que estos índices craneales sirven para determinar el fenotipo racial del individuo, ya que su resultado prominente fue que el principal tipo de cráneo observado era el braquiocefálico y, en menor medida, el mesocefálico. Es decir, en una población tailandesa, el índice craneal prominente es el braquicefálico, propio de poblaciones de origen mongoloide, y en menor medida se hayan cráneos mesocefálicos, propios de poblaciones caucasoides; esto último podría inferirse que es producto de la mezcla y variabilidad actuales en los sujetos.

En concordancia, Matsumura et al. ^20^ evaluaron osamentas encontradas en la localidad de Gua Harimau (Sumatra, Indonesia) y hallaron que esta población comparte orígenes antropológicos con poblaciones australianas, taiwanesas y chinas, es decir, los índices craneales braquicefálicos y mesocefálicos son compartidos por todas estas poblaciones en la actualidad.

En síntesis, con estos estudios se demuestra la variabilidad poblacional que existe actualmente, por lo que hoy en día determinar con exactitud la incidencia racial es bastante arduo. Por ello, cuando existe la necesidad de examinar restos óseos y osamentas, se busca determinar no solo el grupo étnico, sino también el sexo y la edad, caracteres que ayudan a la individualización del sujeto.

### Estimación de la edad

Habitualmente, para la estimación de edad mediante rasgos craneofaciales se evalúa el desarrollo de las estructuras craneales. Esto como consecuencia de que el crecimiento de estos rasgos anatómicos es simétrico y alométrico, es decir, cada estructura crece independiente una de la otra, gracias a estímulos recibidos de la función respiratoria y la deglución; además, los movimientos masticatorios estimulan el crecimiento craneofacial y el desarrollo dental. Adicionalmente, la fusión de suturas craneales, el crecimiento vertebral cervical y el crecimiento mandibular constituyen un gran aporte en el análisis rutinario de estimación de edad cronológica ^21^.

En la actualidad, la estimación de la edad es elemental no solo desde el punto de vista antropológico forense, sino también desde el punto de vista ortodóntico. El análisis del desarrollo craneofacial es de utilidad para la planeación de tratamientos oportunos y diagnósticos de la oclusión. Así lo demuestran estudios como el de Ramírez et al. ^22^, quienes buscaron determinar la correlación de los estadios de maduración de las vértebras cervicales según la edad cronológica en niños y adolescentes. En este estudio, se evaluaron 93 imágenes de radiografías lateral de cráneo, de pacientes entre 6 y 17 años. Se evaluó el estadio de maduración de las vértebras cervicales utilizando el método descrito por Baccetti et al. y para cada estadio se evaluaron los estadísticos descriptivos de la edad.

En el mencionado estudio, se concluyó que existe una relación estrecha entre el estado de maduración de vertebral cervical y la edad cronológica, aunque exista un margen de error ya que la edad cronológica en relación con la edad ósea puede variar debido a factores ambientales o genéticos. De esta manera, se evidencia la significancia del desarrollo de las cervicales en la determinación de la edad desde el punto de vista odontológico clínico y antropológico.

**Figura 2:**
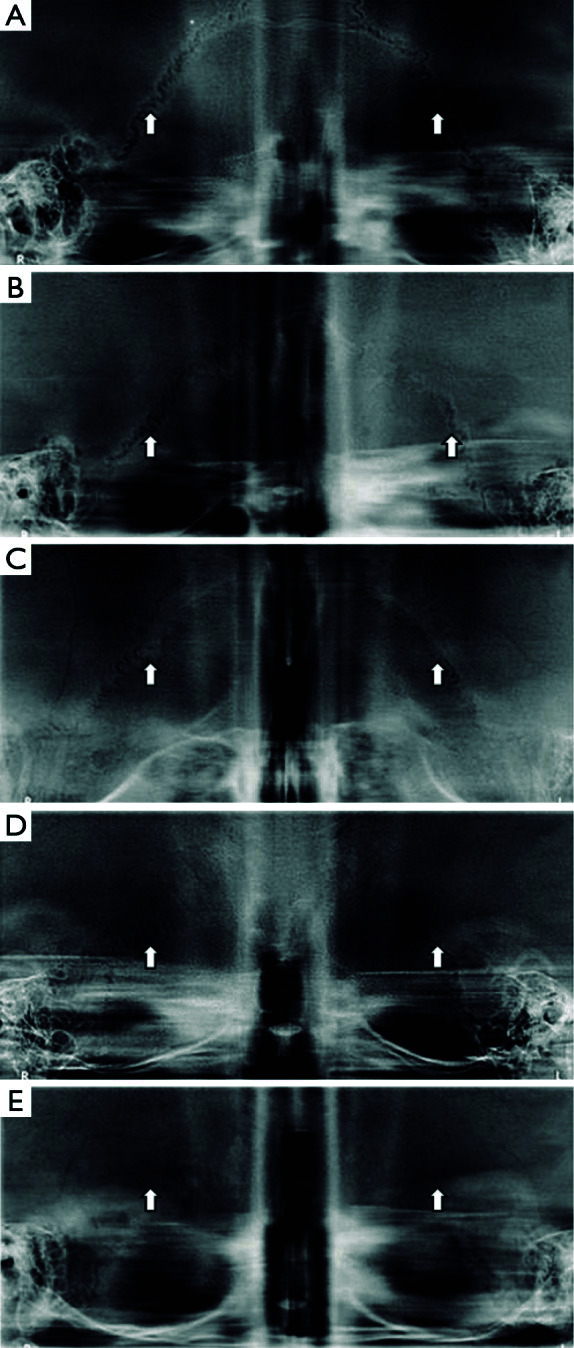
Escalas de cierre de la sutura lambiodea (tomado de Chandra S et al.)

En otro orden de ideas, el trabajo de Chandra et al. ^23^ reconoce la importancia del desarrollo craneal para la estimación de edad. En su investigación, se estudia con detalle el cierre de la sutura lamboidea y su relación con la edad cronológica. Para el estudio, se analizó la radiografía craneal de 85 sujetos, a quienes se colocó al revés, en un sistema panorámico y cefalométrico Kodak 8000C Digital, y se les tomó una radiografía “panorámica”. La sutura lamboidea se observa claramente en la Figura 2.

En conclusión, el análisis radiográfico de estos investigadores determinó que la sutura lamboidea y su cierre son de gran ayuda para estimar la edad un individuo desde el punto de vista antropológico, resaltando la importancia de los rasgos craneales para la determinación de la edad.

Dentro de este marco, Vodanović et al. ^24^ evaluaron la estimación de edad en restos esqueléticos arqueológicos mediante cuatro métodos no invasivos, siendo uno de estos el examen de la sutura palatina. Seleccionaron 192 cráneos encontrados en las excavaciones de la catedral de Santa Teresa de Ávila en Požega (Croacia) y que, según estudios realizados en el 2004, databan de los siglos XVIII y XIX. Determinaron el grado de obliteración de la sutura definiéndola en porciones visibles y no visibles de la misma. Si el cierre u obliteración estaba completo, se consideró que la persona tenía alrededor de 35 años; si la sutura estaba parcialmente abierta o abierta, se consideró que el individuo tenía menos de 35 años al momento de la muerte.

### La antropología dental

Desde hace algunos años, el estudio de la morfología dental ha tenido bastante auge para la determinación del grupo étnico, la edad y el sexo. Se analiza la morfología del diente a través de la estadística para describir la variabilidad entre poblaciones, su origen, costumbres, historia, etc. La estructura dental, coronal y radicular permite establecer relaciones estructurales entre poblaciones; de esta manera, la rama de la antropología que se encarga de este análisis es la denominada antropología dental ^25^^,^^26^.

Un ejemplo claro de este tipo de estudios es el realizado por Valera et al. ^27^, quienes analizaron micro, macro y radiográficamente lo que parecía ser la corona de un incisivo en desarrollo en el sitio arqueológico Playa Chuao, y concluyeron que correspondía a un incisivo central superior deciduo izquierdo, perteneciente a un individuo de sexo femenino en etapa de crecimiento perinatal, que presentaba el rasgo particular de “diente en forma de pala” en su cara palatina, indicador utilizado para la asignación de la ancestría amerindia/mongoloide.

Los “dientes en pala” se caracterizan por la presencia de rebordes proximales muy desarrollados en la cara lingual o palatina, por lo que la extensión lingual de estos rebordes genera una concavidad de distinta profundidad a la normal; a esta concavidad se le denomina fosa lingual o palatina ^28^.

En este marco, Moreno et al. ^29^ buscaron determinar la frecuencia y variabilidad del tubérculo de Carabelli en los primeros molares superiores permanentes de nueve grupos étnicos contemporáneos del suroccidente colombiano. Para ello, los autores recabaron información al respecto en dos estudios y cuatro bases de datos para posteriormente diseñar una matriz de distancias biológicas entre grupos étnicos. De este estudio, resultó que el rasgo anatómico está casi ausente en los grupos étnicos que consideraron, excepto en la población afrocolombiana de la localidad de Tumaco. En vista de este hallazgo, fue necesario evaluar otros rasgos anatómicos dentales como las fosas y expresiones cuspídeas para las poblaciones mestizas caucasoides y otros afrocolombianos donde eran bastante observables.

A partir de lo mencionado, se concluyó que los mestizos caucasoides de Cali, los afrodescendientes de Puerto Tejada y los indígenas nasa estaban asociados con poblaciones mongoloides, mientras que los afrodescendientes de Villa Rica, Guapi y Tumaco eran asociados a poblaciones caucasoides.

De forma similar, Musinguzi et al. ^30^ investigaron la influencia de la práctica ebiino y su relación con la morfología orofacial en infantes de una población rural en Uganda. Esta práctica, también conocida como “extracción de diente falso”, consiste en la enucleación de brotes dentales del canino superior primario al considerar que su remoción cura enfermedades infantiles como fiebres, resfriados y diarrea. 

Se pudo concluir que esta práctica, a largo plazo, ocasiona en poblaciones rurales africanas, especialmente de Uganda, agenesias o defectos en el diente permanente de reemplazo o época tardía de su erupción, reducción del tamaño del arco dental y, lógicamente, daño oclusal a la dentición permanente que ocasiona maloclusiones. Esta práctica, aunque aparentemente iatrogénica, orienta en el análisis craneofacial y la identificación de individuos procedentes de estas poblaciones, ya que las modificaciones de los rasgos anatómicos son irreversibles y distinguen a sujetos provenientes de estas localidades.

De igual manera, Roksandic et al. ^31^ encontraron reducciones coronales de incisivos centrales superiores, lo que constituye una práctica propia del continente africano, en poblaciones caribeñas, específicamente en locaciones como Canímar Abajo, en Cuba. La reducción consistía en disminución de la cara mesial de la unidad dentaria en un ángulo de 45° de dirección mesiodistal, lo que expone la dentina a través del segmento modificado. El hallazgo ocurrió al estudiar material óseo hallado en excavaciones en Canímar Abajo (Cuba), el cual fue exhibido posteriormente en el Museo Montané de la Universidad de la Habana.

**Figura 3:**
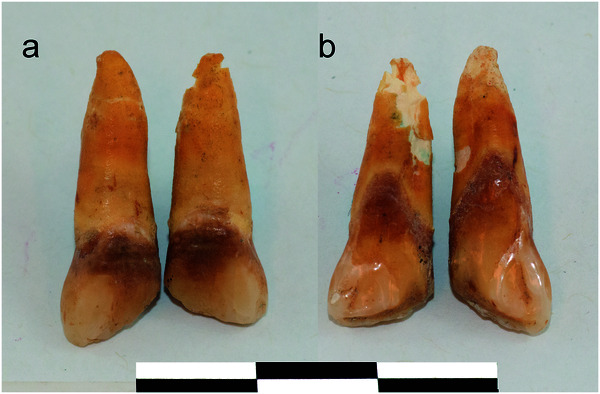
Incisivos centrales modificados (Tomado de Roksandic at al.)

Esto demuestra, antropológicamente, que la morfología dental sirve para la evaluación de la migración poblacional y la ubicación taxonómica de un individuo desde el punto de vista antropológico, así como la identificación de su ancestría y hasta su lugar de procedencia (Figura 3).

En relación con lo mencionado, en Sudamérica, revisiones como la de Fonseca et al. ^32^ señalan que la antropología dental ha servido para el estudio del origen y el poblamiento del continente, ya que en la población sudamericana se observa una tendencia a morfología dental “mongoloide”, ya que existen cuadros coronarios japoneses, indios americanos y esquimales bastante similares. Además, se evidencia que los rasgos dentales de las naciones sudamericanas son las más variadas y completas desde el punto de vista biológico, como consecuencia de sus orígenes genéticos y los fenómenos migratorios a nivel mundial.

En resumen, la antropología dental brinda un aporte a la antropología forense de mucho valor para el análisis de restos óseos u osamentas, y su identificación. El uso de caracteres morfológicos dentales para la determinación de la ancestría o incidencia racial, combinado con la observación y el análisis de rasgos craneofaciales, es de gran utilidad para la determinación del origen americano, europeo, asiático o africano de un sujeto ^33^^,^^34^.

## DISCUSIÓN

Para la determinación del sexo, el grupo étnico y la edad existen métodos tradicionales bien estudiados. Actualmente, herramientas como los estudios radiográficos brindan información bastante confiable y son de gran ayuda para el análisis de rasgos con tal fin. De hecho, se podría decir que la radiografía dentofacial es esencial actualmente en la identificación antropológica y odontológica forense.

Recientemente, se han realizado estudios bastante llamativos que estudian caracteres dentales con fines identificativos. Aunque estos no necesariamente utilizan la radiografía craneofacial ni la observación clínica, constituyen un aporte bastante significativo en la búsqueda de la identidad de un sujeto. Uno de ellos, el de Stewart et al. ^35^, evaluó la determinación del sexo mediante la presencia péptidos en el esmalte dental. El procedimiento consistió en la colocación de ácido en una pequeña porción del esmalte para hacer el estudio lo mínimamente invasivo posible, y de esta forma identificar isoformas de amelogenina, una proteína que forma el esmalte y se encuentra unida al cromosoma sexual. La evaluación se realizó mediante la espectrometría de masas por cromatografía líquida de nanoflujo (nanoLC-MS). Se encontró que la forma dimórfica de amelogenina Y (péptido AMELY) es la correspondiente al sexo masculino y la forma dimórfica de amelogenina X (péptido AMELX) corresponde al sexo femenino. Demostrando la confiabilidad de este método para la determinación del sexo en hallazgos dentarios.

Otro estudio novedoso es el de Baker et al. ^36^, quienes analizaron si los cambios cuantitativos observables en el complejo dentino pulpar están relacionados con la edad y si estos sirven para determinarla. Evaluaron 120 dientes extraídos de distintos grupos etarios, los cuales descalcificaron y sometieron a estudios histopatológicos rutinarios, sometiéndolos a tinciones como hematoxilina, eosina y rojo picrosirius, para medir el número de odontoblastos basales, el diámetro basal y el grueso de las fibras colágenas. Así, observaron una reducción en el número de odontoblastos y del diámetro basal en dientes que pertenecieron a sujetos de edad avanzada, así como un aumento en el grosor de las fibras colágenas, lo que indica una correlación directa de las modificaciones del complejo dentino pulpar con la edad. Por lo tanto, el estudio demostró ser bastante confiable para el análisis antropológico y odontológico de la determinación de la edad.

Por último, se puede mencionar el análisis de Sinthubua et al. ^37^, quienes evaluaron la determinación del sexo mediante el análisis de tres longitudes de la sutura intermaxilar, a saber, anterior, transversa y posterior, mediante la cantidad de pixeles obtenidos de la fotografía digital de estas suturas. Se evaluaron 190 huesos de una población tailandesa (96 masculinos y 94 femeninos) y se encontraron diferencias significativas en la longitud de las suturas entre un sexo y otro, lo que manifestó el dimorfismo sexual en este rasgo anatómico. Los autores destacan que este tipo de rasgos es utilizado tradicionalmente para la determinación de la edad, pero su estudio en este caso demuestra su utilidad antropológica para la determinación del sexo.

## CONCLUSIONES

Es importante la interdisciplinariedad entre la labor del odontólogo y el antropólogo forense para la identificación de restos óseos u osamentas; de hecho, se puede considerar que la determinación de la edad y la sexo son unas de las tareas más arduas para estos profesionales.

El uso de herramientas como la radiografía o la digitalización de estructuras y rasgos anatómicos juega un papel clave en la identificación antropológica y odontológica forense. En la actualidad, el análisis de la mandíbula y su morfología mediante estas herramientas ha probado ser bastante útil para la determinación del sexo.

En el caso del grupo étnico, desde el punto de vista antropológico forense, actualmente se torna un poco más compleja su determinación, gracias a la variabilidad fenotípica existente. Se pueden establecer algunos rasgos característicos de cada nación, así como evaluar las modificaciones anatómicas que se realizan los individuos producto de cada una de las culturas y, de esta forma, identificar la ancestría de una forma bastante certera.

Por último, la determinación de los grupos etarios es de mucha importancia no solo desde una visión forense e identificativa, sino también para la planeación de tratamientos ortodónticos y diagnósticos. Esta puede realizarse mediante muchos métodos clínicos y radiográficos, que incluyen el estudio de estructuras y rasgos tanto anatómicos como dentales que permiten establecer con bastante certeza la edad de un individuo.

## Fuente de financiamiento:

Investigación autofinanciada
